# Therapeutic efficacy of tranexamic acid on traumatic brain injury: a systematic review and meta-analysis

**DOI:** 10.1186/s13049-024-01188-z

**Published:** 2024-03-07

**Authors:** Jia-Xing Song, Jian-Xiang Wu, Hai Zhong, Wei Chen, Jian-Chun Zheng

**Affiliations:** Department of Emergency, The Second Hospital of Jiaxing: The Second Affiliated Hospital of Jiaxing University, Jiaxing, 314000 Zhejiang Province China

**Keywords:** Tranexamic acid, Traumatic brain injury, Therapeutic efficacy, Meta-analysis

## Abstract

**Objective:**

Tranexamic acid (TXA) demonstrates therapeutic efficacy in the management of traumatic brain injury (TBI). The objective of this systematic review and meta-analysis was to evaluate the safety and effectiveness of TXA in patients with TBI.

**Methods:**

The databases, namely PubMed, Embase, Web of Science, and Cochrane Library databases, were systematically searched to retrieve randomized controlled trials (RCTs) investigating the efficacy of TXA for TBI from January 2000 to November 2023.

**Results:**

The present meta-analysis incorporates ten RCTs. Compared to the placebo group, administration of TXA in patients with TBI resulted in a significant reduction in mortality (*P* = 0.05), hemorrhage growth (*P* = 0.03), and volume of hemorrhage growth (*P* = 0.003). However, no significant impact was observed on neurosurgery outcomes (*P* = 0.25), seizure occurrence (*P* = 0.78), or pulmonary embolism incidence (*P* = 0.52).

**Conclusion:**

The administration of TXA is significantly associated with reduced mortality and hemorrhage growth in patients suffering from TBI, while the need of neurosurgery, seizures, and incidence of pulmonary embolism remains comparable to that observed with placebo.

## Introduction

The substantial global public health concern posed by traumatic brain injury (TBI), characterized by elevated rates of mortality and disability, necessitates its recognition as a prominent societal issue deserving significant attention [1]. The condition poses a significant threat to the health and quality of life of young adults, particularly in low- and middle-income countries where disability and mortality rates are disproportionately high [2]. The secondary brain injury resulting from progressive intracranial hemorrhage, cerebral edema, elevated intracranial pressure, and subsequent cerebral ischemia constitutes the primary etiologies of morbidity and mortality following TBI [3–5]. Post-traumatic coagulation dysfunction, occurring in around one-third of patients with brain injury, is responsible for the aggravation of secondary brain injury and greatly escalates the risk of mortality by a factor of ten [3, 6, 7].

The antifibrinolytic agent tranexamic acid (TXA), commonly administered to trauma patients, exhibits hemostatic activity [8]. The synthetic lysine derivative TXA competes for occupancy of the lysine-binding site on plasminogen, thereby impeding its interaction with fibrin and thwarting thrombus formation [9]. The potential role of TXA in reducing hematoma size and preventing secondary brain injury has prompted its proposition as a therapeutic intervention with the capacity to optimize clinical outcomes in patients with TBI.

The etiology of TBI encompasses a multitude of factors, including armed conflicts, vehicular accidents, falls from significant heights, and other related causes. Receiving timely intervention following TBI plays a pivotal role in improving patient prognosis and optimizing treatment costs. The management of TBI patients in eligible regions and hospitals should be carried out by multidisciplinary trauma teams to optimize patient survival rates [10]. The role of fibrinolysis as a pivotal pathophysiological component in post-traumatic coagulation dysfunction has been firmly established, with heightened fibrinolysis serving as a significant determinant for mortality among trauma patients [11]. The anti-fibrinolytic effect of TXA is achieved by competitively inhibiting the formation of plasmin. When TXA binds to lysine-binding domains exposed on plasminogen, it induces a decrease in plasmin levels, thereby impeding clot lysis [12].

The existing meta-analyses in this field are limited by factors such as small sample sizes or outdated data, and a majority of them lack comprehensive analysis on the neurological outcomes and prognosis of patients. The aim of this study was to assess the urgency of administering TXA in patients with traumatic brain injury and identify clinical indicators that can be enhanced following TXA administration, thereby enhancing survival rates and providing prognostic benefits to patients. The present study conducted a comprehensive systematic review and meta-analysis of randomized controlled trials (RCTs) investigating the use of TXA in the management of TBI, aiming to provide evidence-based guidance for clinical practice by elucidating both the potential benefits and drawbacks associated with TXA administration.

## Materials and methods

### Search strategy

The study was registered on PROSPERO (CRD42023494345). The literature review and meta-analysis were conducted in accordance with the PRISMA statement as well as the guidelines outlined in the Cochrane handbook for Systematic reviews [13]. Take PubMed as an example, as follows: (Craniocerebral trauma OR Craniocerebral injury OR Severe craniocerebral injury OR Severe traumatic brain injury OR Severe brain injury OR HeadInjury) AND (Tranexamic acid). Two authors independently searched the PubMed, Embase, Web of Science, and Cochrane Library databases for RCTs relevant studies published from January 2000 to November 2023. No language restrictions were applied, and the search involved use of studies with inclusion of participants. The reference lists of all retrieved articles were meticulously scrutinized to further identify potentially relevant studies.

### Inclusion and exclusion criteria

Any studies that met the following criteria were considered: (1) The studies were conducted in patients suffering from TBI; (2) TXA was administered via various routes and at different doses following admission; and (3) The study incorporated at least one outcome measure that is pertinent to the research.

Articles that met the following criteria were not considered: (1) Not all patients were afflicted with TBI; (2) Letters, review articles, case reports, and any other stylistic features that fail to provide comprehensive trial information; and (3) non-RCTs.

### Data extraction and outcome measures

After removing duplicates, the titles and abstracts were independently assessed by two investigators based on the predefined inclusion criteria for conducting a meta-analysis. Following the initial screening of titles and abstracts, articles that presented unresolved uncertainties underwent a comprehensive evaluation conducted independently by two investigators. Any discrepancies were resolved prior to reaching a final consensus. The primary outcome measures included mortality, hemorrhage growth, neurosurgery, seizures and pulmonary embolism (PE).

### Literature quality evaluation

The assessment of risk bias and literature quality was conducted by two reviewers in accordance with the Cochrane Evaluation Manual Version 5.3. The evaluation tool included seven items: random sequence generation, allocation concealment, blinding of participants and personnel, blinding of outcome assessment, incomplete outcome data, selective reporting, and other bias. Each assessed as “low risk”, “unclear risk”, “high risk”.

### Statistical analysis

The Meta-analysis was conducted using Review Manager 5.4 software. The weighted mean differences (WMD) and risk ratios (RR) were employed for the comparison of continuous and dichotomous variables. The heterogeneity among different studies was assessed using the Chi-square test, and statistical significance was defined as P < 0.05. The I^2^ statistic was employed to quantify heterogeneity, utilizing the random-effects model in case of observed heterogeneity among studies, and resorting to the fixed-effects model otherwise. The sensitivity analysis was conducted on high-quality studies, utilizing Stata 17.0 to identify potential publication bias.

## Results

### Literature search, study characteristics and quality assessment

**Fig. 1:**
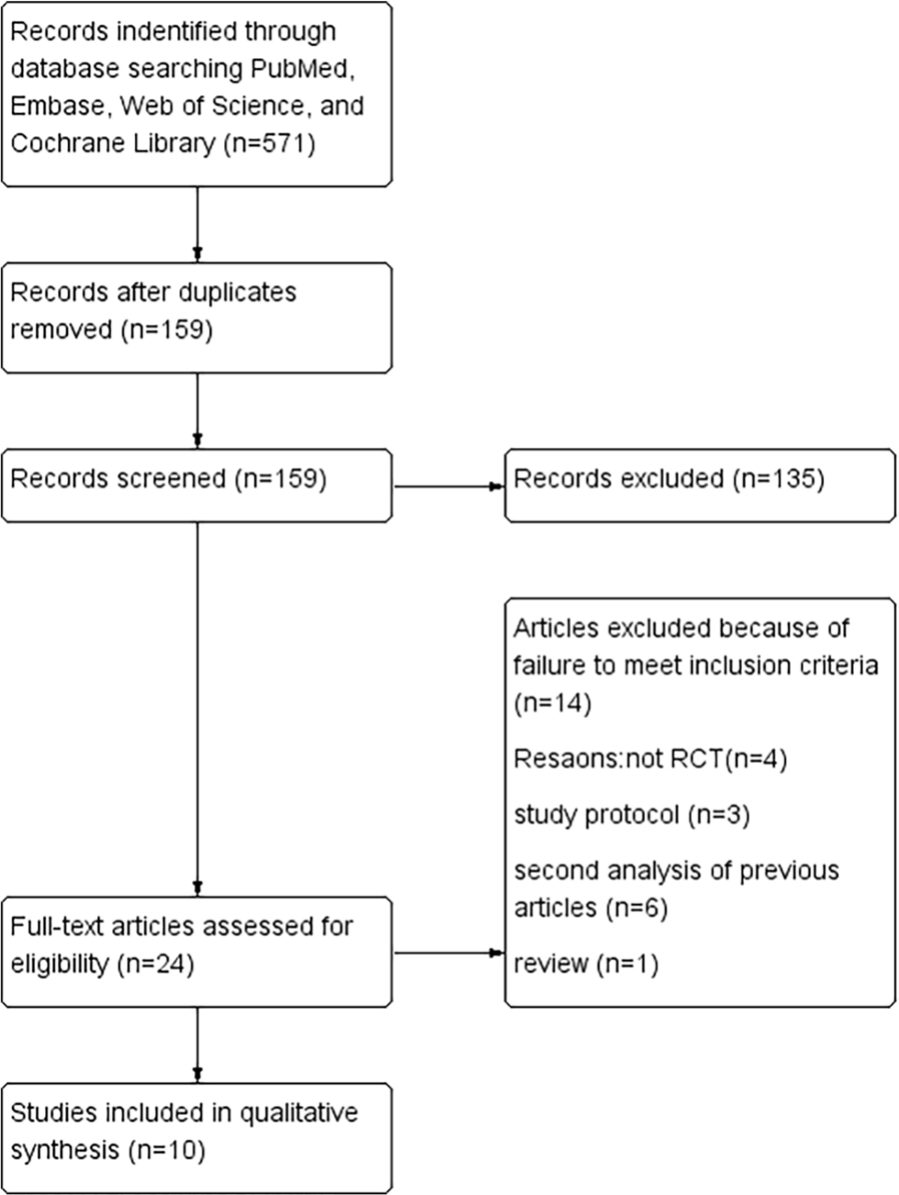
Flowchart of the search strategy

The process of study selection is summarized in Fig. 1. The initial search of databases yielded a total of 571 publications. After reviewing the publications, the full-text of 24 studies underwent further assessment. Out of these, 14 papers failed to meet the predetermined criteria and were subsequently excluded. Ultimately, a total of 10 studies [14–23] were deemed suitable for inclusion in the meta-analysis. Relevant studies were not found through manual screening of the reference lists of these ten studies.

The baseline characteristics of ten eligible RCTs are presented in Table 1. The detailed approach to the treatment of traumatic brain injury with tranexamic acid is summarized in Table 1.

**Table 1:** Characteristics of included studies

Author	Tranexamic acid group					Control group				
	Sample size	Mean age (years)	Gender (male/female)	Methods	GCS	Sample size	Mean age (years)	Gender (male/female)	Methods	GCS
Chakroun‑Walha 2018	96	44 ± 20	88/8	Intravenous tranexamic acid was administered as soon as possible, with a first dose of 1 g in 100 mL of normal saline in 10 min and then with a maintenance dose of 1 g per 500 mL of normal saline for 8 h	9 ± 5	84	39 ± 18	74/10	Matched placebo	10 ± 5
CRASH-3 2019	4649	41.7 ± 19	3742/906	Tranexamic acid (loading dose 1 g over 10 min, then infusion of 1 g over 8 h)	Mild (13–15):1307 Moderate (9–12):1557 Severe (3–8):1757	4553	41.9 ± 19	3660/893	Matched placebo	Mild (13–15):1262 Moderate (9–12):1524 Severe (3–8):1732
Ebrahimi 2019	40	40.39 ± 18.27	33/7	Infusion of the drug as a loading dose (combination of 1 g of TXA with 100 ml of 0.9% normal saline and intravenous infusion over 10 min) and maintenance dose (combination of 1 g of TXA with 500 ml of 0.9% normal saline and an intravenous infusion over a period of 8 h)	Mild (13–15):8 Moderate (9–12):4 Severe (3–8):24	40	24.47 ± 7.11	35/5	Matched placebo	Mild (13–15):10 Moderate (9–12):3 Severe (3–8):23
Fakharian 2017	74	42.3 ± 18.3	67/7	Intravenous tranexamic acid was administered with the first dose of 1 g in 100 mL of normal saline in 10 min and then with a maintenance dose of 1 g per 1000 mL of normal saline for 8 h	12.7 ± 2.83	75	39.3 ± 18.1	66/9	Matched placebo	11.65 ± 3.71
Jokar 2017	40	35.4 ± 14.6	32/8	Intravenous tranexamic acid (a bolus of 1 g in 100 mL 0.9% NaCl over 10 min followed by a continuous infusion of 1 g in 500 mL 0.9% NaCl over 8 h)	< 8	40	36.2 ± 14.9	28/12	Matched placebo	< 8
Mousavinejad 2020	20	54.8 ± 19.1	14/6	Infusion in the form of loading dose (combination of 1 g TAX with 500 ml of 0.09% normal saline and intravenous Infusion within 10 min) and maintenance dose (combination of 1 g TAX with 500 ml of 0.09% normal saline and Intravenous Infusion within 8 h)	Mild (13–15):1 Moderate (9–12):5 Severe (3–8):13	20	55.1 ± 18.1	12/8	Matched placebo	Mild (13–15):2 Moderate (9–12):3 Severe (3–8):14
Perel 2012	133	36 ± 14	111/22	Tranexamic acid (loading dose 1 g over 10 min, then infusion of 1 g over 8 h)	Mild (13–15):63 Moderate (9–12):25 Severe (3–8):45	137	37 ± 14	117/20	Matched placebo	Mild (13–15):58 Moderate (9–12):34 Severe (3–8):45
Rowell 2020	312	30.5 ± 11.5	227/85	1-g IV tranexamic acid bolus in the out-of-hospital followed by a 1-g tranexamic acid IV infusion initiated upon hospital arrival and infused over 8 h	Mild (13–15):14 Moderate (9–12):129 Severe (3–8):169	309	29 ± 11.1	233/76	Matched placebo	Mild (13–15):8 Moderate (9–12):115 Severe (3–8):186
	345	30.5 ± 11.1	255/90	2-g IV tranexamic acid bolus in the out-of-hospital setting followed by a placebo infusion	Mild (13–15):9 Moderate (9–12):159 Severe (3–8):177					
Safari 2021	47	36.2 ± 15.1	40/7	Initial dosage of 1 g TXA within the first 3 h of admission, followed by 1 g every 6 h for 48 h	11.1 ± 2.9	47	36.4 ± 14.1	38/7	Matched placebo	11.1 ± 3.0
Yutthakasemsunt 2013	120	34.8 ± 16.0	103/17	Tranexamic acid (loading dose of 1.0 g over 30 min followed by a maintenance dose of 1.0 g infused over eight hours)	Moderate (9–12):52 Severe (4–8):68	118	34.1 ± 15.3	107/11	Matched placebo	Moderate (9–12):47 Severe (4–8):71

**Fig. 2:**
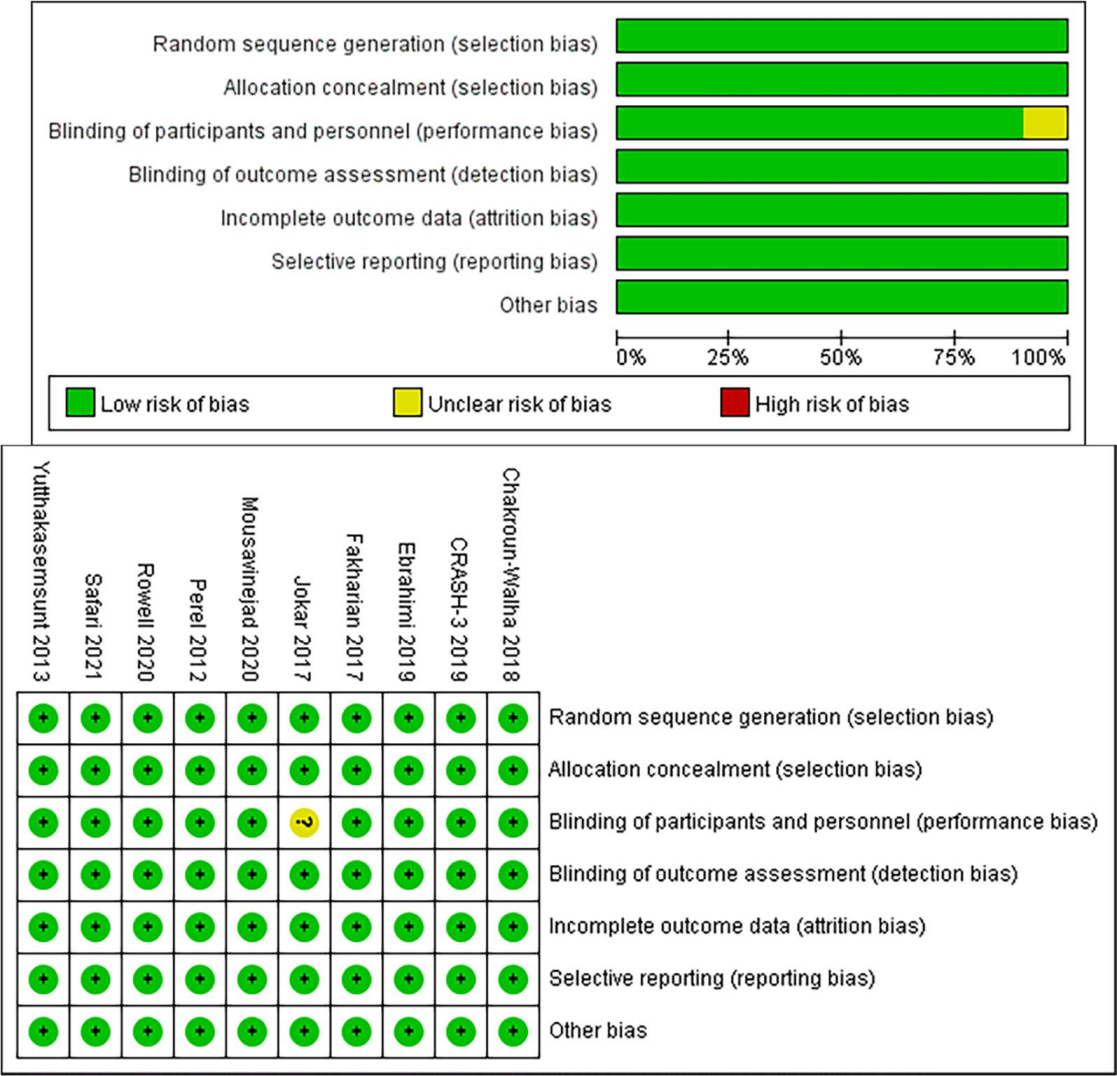
Quality evaluation chart

Among the ten RCTs, eight studies have reported on mortality [14–17, 19–21, 23], hemorrhage growth has been reported in four studies.[17, 20, 22, 23], four studies have reported the volume of hemorrhage growth [17, 18, 20, 22], five studies have reported the need for neurosurgery [14, 17, 20, 21, 23], Seizures has been reported in in two studies [15, 21], three studies have reported pulmonary embolism [14, 15, 21]. The quality of nine studies is high, while one study exhibits moderate quality. The quality assessments for the eligible studies are presented in Fig. 2.

### Results of meta-analysis

#### Mortality

**Fig. 3:**
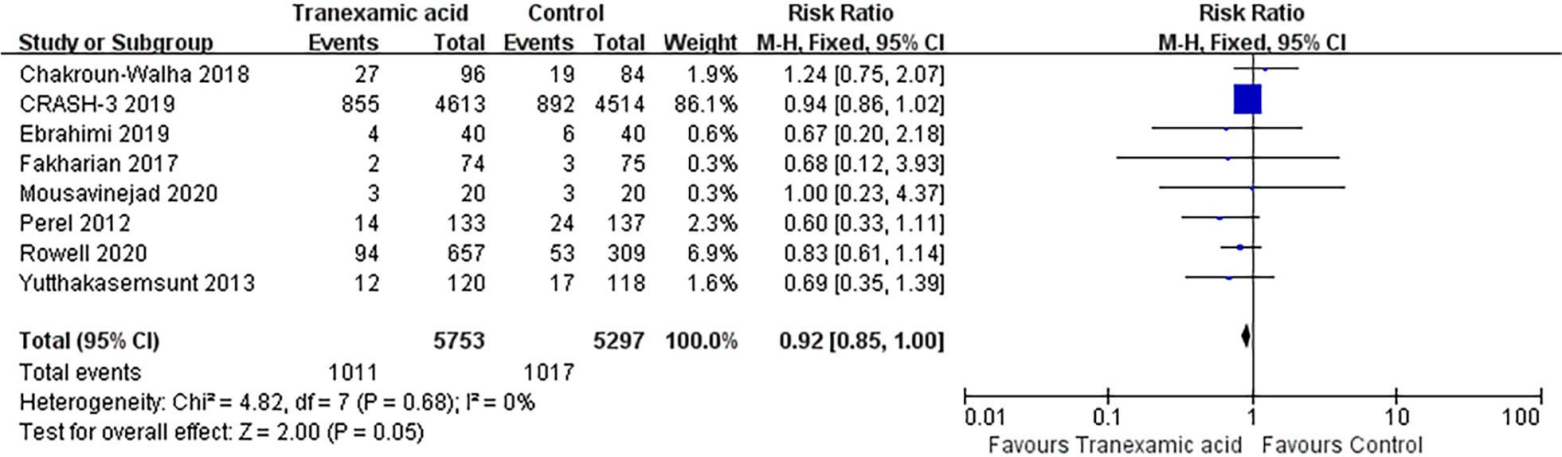
Meta-analysis of pooled data on mortality

The fixed-effect model is employed for the analysis of mortality, and eight included RCTs report this outcome measure. The administration of TXA leads to a significantly reduced mortality rate compared to the placebo group in patients with TBI (RR = 0.92; 95% CI = 0.85 to 1.00; *P* = 0.05, Fig. 3).

#### Hemorrhage growth

**Fig. 4:**
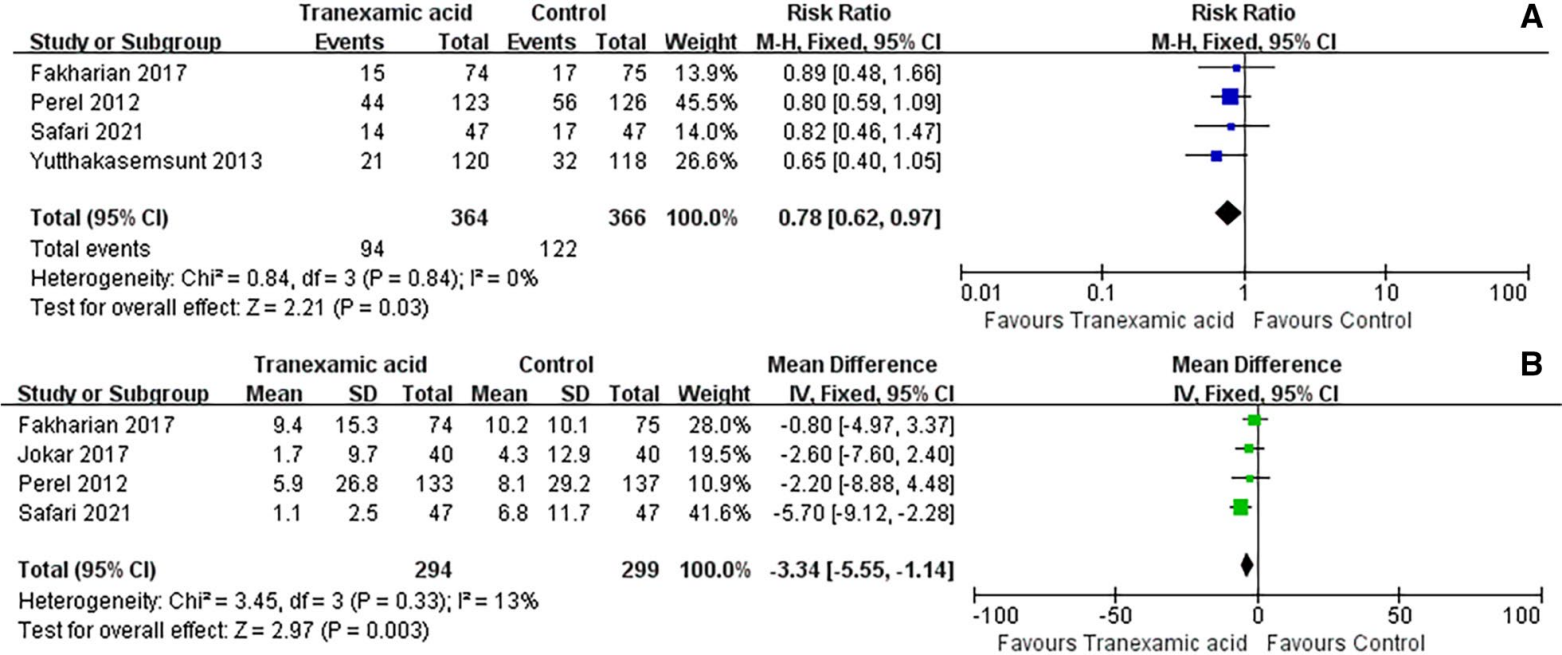
Meta-analysis of pooled data on hemorrhage growth (**A**), volume of hemorrhage growth (**B**)

The hemorrhage growth was documented in four studies. The intervention of TXA is significantly associated with a substantial reduction in hemorrhage growth, as compared to the placebo group for TBI (RR = 0.78; 95% CI = 0.62 to 0.97; *P* = 0.03; Fig. 4A). Similarly, four studies reported the quantification of hemorrhage growth volume. The analysis results demonstrated that TXA intervention effectively mitigated hemorrhage growth volume in TBI patients compared to the placebo group (MD = -3.34; 95% CI = -5.55 to -1.14; *P* = 0.003; Fig. 4B).

#### Neurosurgery

**Fig. 5:**
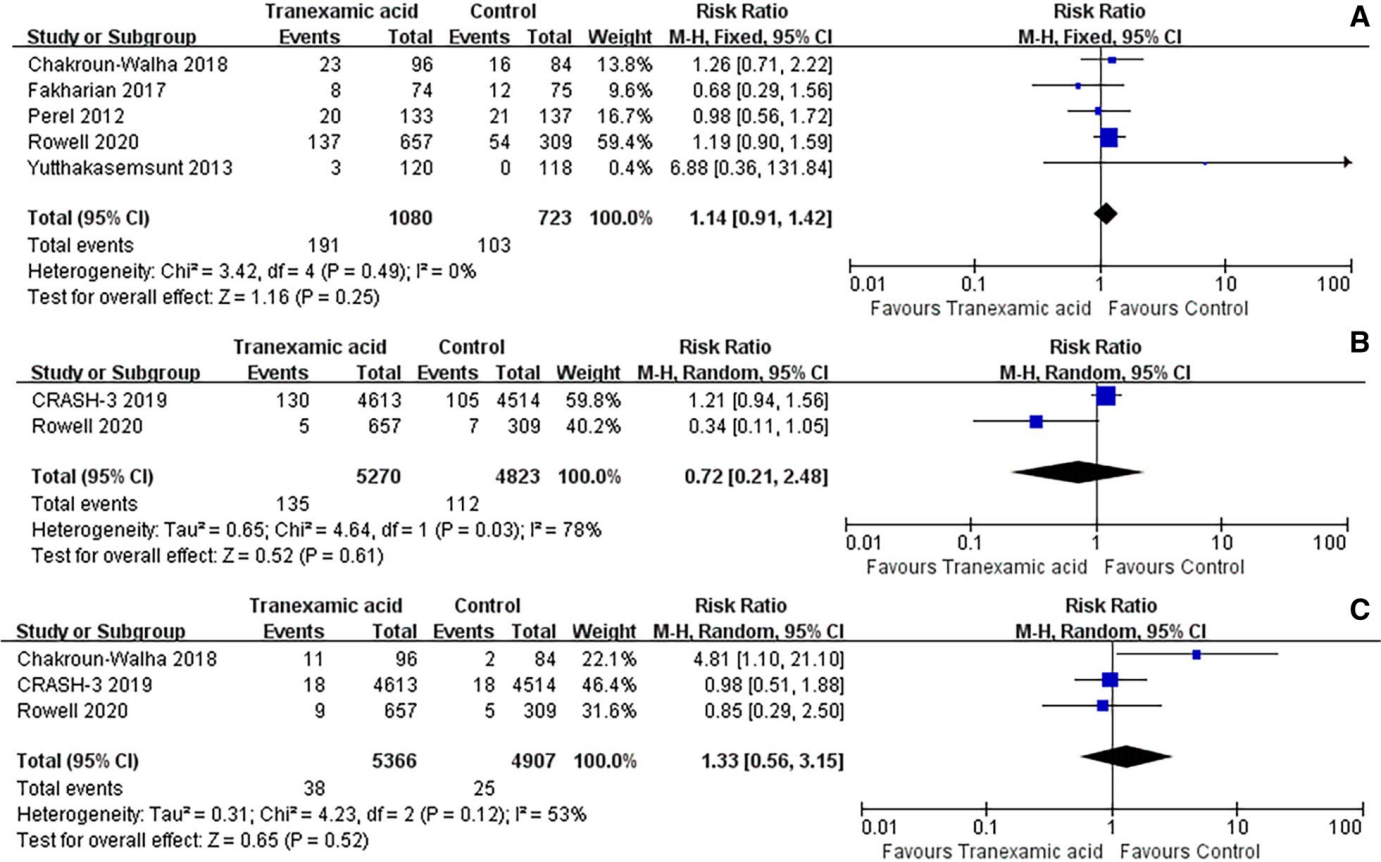
Meta-analysis of pooled data on neurosurgery (**A**), seizures (**B**), PE (**C**)

A total of 5 studies provided data on the requirement for neurosurgery following treatment with TXA. Upon pooling the data for analysis, no statistically significant differences in the need for neurosurgery were observed (RR = 1.14; 95% CI = 0.91 to 1.42; *P* = 0.25; Fig. 5A).

#### Seizures

Seizures were observed at the conclusion of the follow-up period in two conducted studies. No statistically significant difference was observed between the TXA intervention and placebo groups in TBI patients. (RR = 0.72; 95% CI = 0.21 to 2.48; *P* = 0.61; Fig. 5B).

#### Pulmonary embolism (PE)

In the four studies that reported PE. The TXA intervention did not yield any significant difference compared to the placebo groups in TBI patients. (RR = 1.33; 95% CI = 0.56 to 3.15; *P* = 0.52; Fig. 5C).

#### Publication bias

**Fig. 6:**
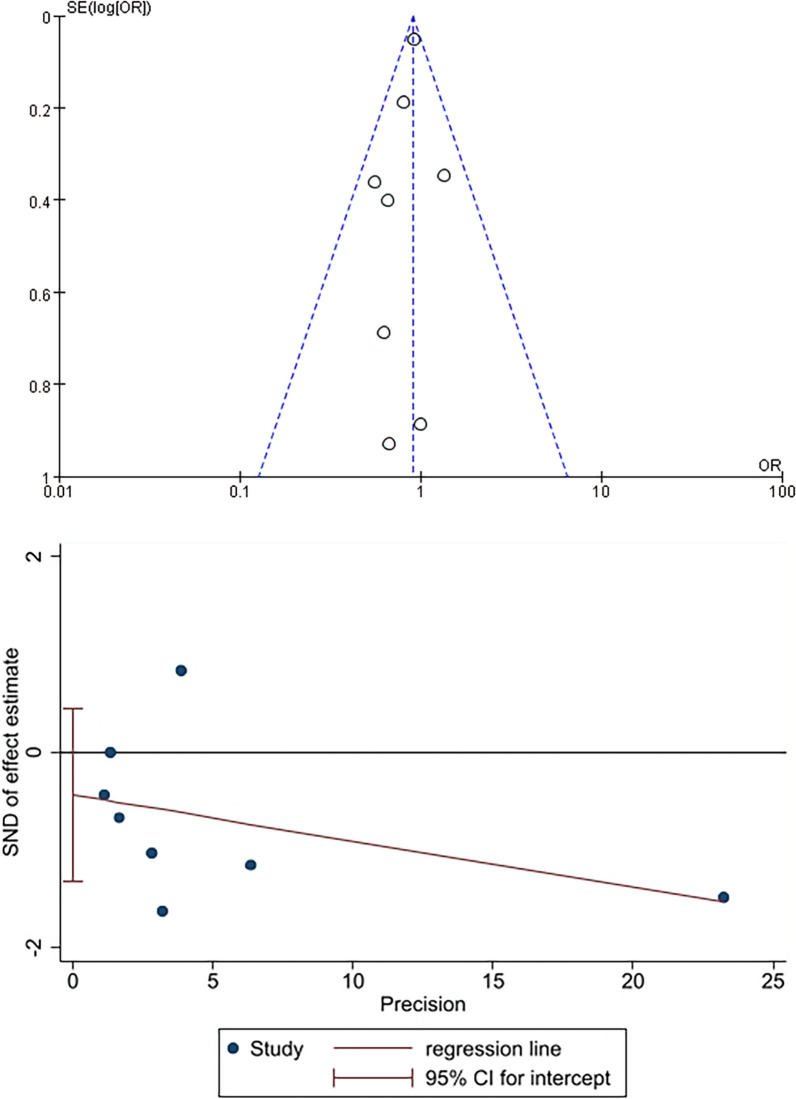
Funnel plot showing symmetrical distribution of studies and the absence of publication bias. The Egger’s test (*P* = 0.271), indicating the absence of publication bias

The funnel plot was employed to evaluate the presence of publication bias. The absence of asymmetry in the funnel plot suggests no indication of publication bias. The results of Egger's test did not reveal any discernible publication bias (*P* = 0.271; Fig. 6).

## Discussion

Given the unalterable nature of primary brain injury following traumatic brain injury (TBI), it becomes imperative to focus on mitigating secondary brain injury in order to optimize the prognosis for patients with TBI. Secondary injuries encompass the expansion of hematoma, development of cerebral edema, elevation of intracranial pressure, occurrence of infection, hypoxia, and coagulopathy. The cerebral tissue contains significant amounts of thromboplastin, which are released into the bloodstream at elevated concentrations following physical trauma to the parenchyma, thereby initiating disruptions in the coagulation process [24, 25]. Additionally, impaired cerebral endothelium activates platelets and initiates the coagulation cascade, resulting in intravascular thrombosis and depletion of coagulation factors [26].

The synthetic compound tranexamic acid (TXA), composed of lysine analogues, functions as a potent inhibitor of plasminogen activation. This mechanism facilitates the preservation of mature fibrin clots and ensures continuous coagulation. The rationale behind the enhanced clinical outcomes observed with TXA treatment in TBI lies in its potential to mitigate secondary brain injury through two mechanisms. Firstly, as an antifibrinolytic agent, TXA may exert its effect by inhibiting fibrinolysis and thereby impeding the progression of intracerebral hemorrhage—a common occurrence in TBI that has been identified as a significant independent predictor [27]. Secondly, through the inhibition of tissue plasminogen activator activity, TXA may also exert a potential role in ameliorating perilesional edema [28].

The CRASH-3 trial has demonstrated the safety of TXA in patients with TBI, and early administration within 3 h of injury significantly reduces mortality associated with head injuries [15]. Hence, the timing of tranexamic acid administration mentioned in the literature predominantly early intervention. The primary outcome for CRASH-3 was modified during the trial, shifting from all-cause mortality to head-injury-related mortality within 28 days following the injury [15]. Due to the inherent subjectivity in the classification of causes of death, this approach is susceptible to classification errors and has the potential to introduce bias into otherwise objective findings [29]. Therefore, the mortality mentioned in the article refers to all-cause mortality, rather than specifically deaths related to head injury.

In this meta-analysis, 10 studies involving 11,299 patients were included. Our meta-analysis suggests that TXA can substantially reduce mortality compared to placebo for TBI. The expansion of intracranial hematoma can lead to an increase in intracranial pressure, cerebral herniation, and even fatality. The synthetic derivative of lysine, TXA, exerts its action by inhibiting fibrinolysis through the blockade of lysine binding sites on plasminogen. This mechanism attenuates fibrinolysis levels during the early stages following injury, thereby mitigating the expansion of intracranial hematoma. The nested analysis of CRASH-3 revealed that [30] individuals who succumbed to craniocerebral injury exhibited a median time-adjusted increase in intracranial hemorrhage of 37 ml/h, whereas those who perished from non-craniocerebral injury experienced an increase of only 11 ml/h. The meta-analysis conducted by July [31] demonstrated that treatment with TXA significantly reduced mortality (RR = 0.92, 95% CI 0.88–0.97, *P* = 0.002) and hematoma expansion rate (RR = 0.79, 95% CI 0.64–0.97, *P* = 0.03) compared to intravenous placebo. Additionally, tissue injury and the subsequent release of inflammatory mediators following TBI can induce endothelial dysfunction, while the released inflammatory mediators can further contribute to vascular endothelial cell injury and depletion of coagulation factors. The activation of the inflammatory response and sympathetic adrenal system can induce an upregulation of extracellular proteolytic enzymes, resulting in shedding of the polysaccharide coating. These processes collectively contribute to an augmentation in blood–brain barrier permeability, potentially serving as underlying mechanisms for the development of intracerebral hemorrhage and brain edema following TBI [32]. Anderson et al. [33] demonstrated that early administration of TXA in patients with moderate and severe TBI can mitigate the elevation of plasma syndecan-1 and angiopoietin-2, inhibit protease-mediated degradation of vascular polysaccharide coating, and attenuate endothelial injury.

The administration of tranexamic acid, as compared to placebo, is associated with a significant reduction in both the extent and volume of hemorrhage growth in TBI. Hijazi et al. [34] employed a murine model of TBI to investigate the impact of tissue plasminogen activator (tPA) and urokinase plasminogen activator (uPA) gene knockout on persistent intracranial hemorrhage in TBI mice, revealing a significant reduction compared to wild-type mice. The tPA and uPA function as the two primary physiological triggers for initiating the activation of plasminogen. They proposed that TBI-induced endogenous hyperfibrinolysis acts as the primary determinant of hematoma expansion and coagulation abnormalities subsequent to TBI. The principal mediator of fibrinolysis is plasmin, which is generated via the cleavage of circulating plasminogen. The incidence of coagulation dysfunction in patients with TBI can reach up to 60%, primarily attributed to hyperfibrinolysis triggered by the surge of endogenous plasminogen activator [35]. The interaction between plasminogen and plasmin is impeded by TXA, thereby obstructing the activation of plasmin and inhibiting fibrinolysis. The size of hematoma diminishes proportionally with the initiation of TXA treatment at an earlier stage [36].

The findings of our study indicate that there is no statistically significant disparity in the number of patients necessitating neurosurgery following treatment with TXA. The decision to perform the operation may have been influenced by various other factors that could potentially attenuate the impact of TXA treatment in patients with TBI, such as the hematoma's location, patients' vital signs, clinical judgments made by physicians, and so on. The results of our analysis, however, still demonstrate the effective reduction of hematoma expansion and improvement in clinical outcomes by TXA. The achievement of a positive clinical outcome relies on the proper implementation of essential operations, which must be carried out as considered indispensable.

The TXA intervention did not yield a statistically significant difference in seizure occurrence compared to the placebo group among patients with TBI. The structural similarity between TXA and glycine allows for its competitive inhibition of glycine receptors in cortical and spinal cord neurons, as well as γ-aminobutyric acid receptors in cortical and medullary neurons of rats. This dual pathway inhibition can lead to an upregulation of excitatory synaptic stimulation, ultimately precipitating seizure occurrence [37]. The primary and secondary impairments to the blood–brain barrier in patients with TBI may contribute to an increased incidence of seizures associated with TXA. Additionally, the early administration of sedative medications in specific individuals with severe TBI may potentially obscure seizure symptoms.

No statistically significant difference was observed between the TXA intervention and placebo groups in terms of pulmonary embolism among patients with TBI. Theoretically, TXA's antifibrinolytic properties may potentially increase the risk of microvascular thrombosis or even pulmonary embolism (PE) if fibrinolysis is impaired or halted. The presence of PE, which can be induced by various confounding factors such as the interference of suspended red blood cells, plasma, and platelets, should also be considered as a potential confounder. Coagulation dysfunction following TBI is a complex and dynamic process, characterized by diverse phenotypes of dysfunction occurring at varying time intervals. The non-individualized administration of TXA may potentially contribute to the development of PE, although no relevant studies have yet confirmed this assertion. Nevertheless, the early administration of TXA in the immediate aftermath of injury remains a prevalent clinical practice.

The present study still possesses certain limitations. First, the present study incorporated the most comprehensive RCTs conducted to date, despite significant disparities in sample sizes observed across certain studies (exceeding 300 participants). Second, the type and severity of TBI, as well as the duration of TXA administration, varied across each included RCTs, potentially influencing the observed outcomes. Third, previous studies have presented incomplete raw data, potentially introducing biases that may impact the validity of the findings.

## Conclusion

The present meta-analysis demonstrates the efficacy of tranexamic acid in reducing mortality and hemorrhage growth among patients with traumatic brain injury, while observing no significant impact on the need of neurosurgery, seizures, or incidence of pulmonary embolism. The timely administration of tranexamic acid in patients with traumatic brain injury is pivotal for minimizing hematoma volume. Further robust randomized controlled trials investigating the efficacy and safety of tranexamic acid treatment in traumatic brain injury patients are warranted in future research.
